# A new method to evaluate fluoroscopic system collimator performance

**DOI:** 10.1002/acm2.14536

**Published:** 2024-10-09

**Authors:** Joseph R. Steiner, Courtney K. Morrison, Mayur Vaya, Nicholas Bevins, Jeremy Christophel, Matt Vanderhoek

**Affiliations:** ^1^ Department of Radiology University of Chicago Chicago Illinois USA; ^2^ Department of Diagnostic Radiology and Nuclear Medicine RUSH University Medical Center Chicago Illinois USA; ^3^ Department of Radiology Henry Ford Health Detroit Michigan USA; ^4^ Department of Radiology Maine Medical Center Portland Maine USA

**Keywords:** equipment performance evaluation (EPE), fluoroscopy, quality control

## Abstract

**Introduction:**

Fluoroscopy uses collimators to limit the radiation field size. Collimators are often evaluated annually during equipment performance evaluations to maintain compliance with regulatory and/or accreditation bodies. A method to evaluate and quantify fluoroscopy collimator performance was developed.

**Methods:**

A radiation field and displayed image measurement device consisting of radiopaque rulers and radiochromic film strips was placed on the x‐ray source assembly exit window to evaluate fluoroscopy collimator performance. This method was used to evaluate collimator performance on 79 fluoroscopic imaging systems including fixed C‐arms, mobile C‐arms, mini C‐arms, and radiographic fluoroscopic systems.

**Results:**

The excess length (EL), excess width (EW), and sum EL + EW of the radiation field relative to the displayed image were measured and compared to the limits specified in 21CFR1020.32. Four systems exceeded these limits. Placing the radiation measurement device at the x‐ray source assembly exit window relative to the image receptor cover increased the film exposure rate by a factor up to 14.6. The time required to set up and complete the fluoroscopy collimator performance measurements using this method ranged from 5 to 10 min.

**Conclusions:**

This method provides an easily implemented quantitative measure of fluoroscopy system collimator performance that satisfies regulatory and accreditation body requirements.

## 1 INTRODUCTION

During fluoroscopic imaging procedures, the x‐ray field of view (FOV) is modified using x‐ray attenuating collimators; this improves image quality and reduces radiation dose to patients and staff.^1^, ^2^ Collimator performance is typically assessed during fluoroscopic equipment performance evaluations (EPEs) performed by medical physicists or other trained individuals.^3^ Many institutions perform fluoroscopic EPEs annually as required or recommended by The Joint Commission (TJC),^4^ the American College of Radiology (ACR),^5^ the American Association of Physicists in Medicine (AAPM),^5^, ^6^, ^7^ the International Atomic Energy Agency (IAEA),^8^ and/or various local, state and federal agencies. Several methodologies to evaluate collimator performance have been described.^6^, ^8^, ^9^, ^10^ The simplest method is to ensure the collimator blades are just visible on the displayed image.^6^ Other methods allow for quantitative comparisons of the radiation field and displayed image sizes by placing a radiation field measurement device, including fluorescent screens,^6^, ^9^ film,^6^, ^8^, ^10^ computed radiography plates,^8^ or radiation sensitive rulers,^6^ on the image receptor cover with an appropriate length marking device (e.g., radiopaque rulers).

These techniques suffer from several challenges. Having the collimator blades just visible on the displayed image does not provide quantitative data, requires a level of physicist involvement that may not be possible (e.g., consultants), and in some modern fluoroscopy systems, the detectors turn off at the assumed field edges which may present as collimator blades. When using fluorescent screens, visually examining the screen in‐room increases occupational exposure. This can be mitigated by using video recording devices, but these can be time‐consuming and/or challenging to set up. For fluorescent screens and film, it can be difficult and/or time‐consuming to produce a sufficient air kerma rate at the image receptor cover to visibly see the field or darken the film. Lead sheets can be sandwiched between the image receptor cover and the radiation measurement device to drive the system to a higher technique, but the lead sheets reduce image quality and/or prevent exposure from occurring which can make it challenging to identify the size of the displayed image. Finally, devices such as radiation‐sensitive electronic rulers can be expensive and time‐consuming (if only a single electronic ruler is available, four exposures are required to measure each radiation field edge).

To address these challenges, a method was developed that places the radiation field measurement device on the x‐ray source assembly exit window. The feasibility of this method was demonstrated through analysis of EPEs performed on fluoroscopy systems over a 6‐month period.

## 2 METHODS

**FIGURE 1:**
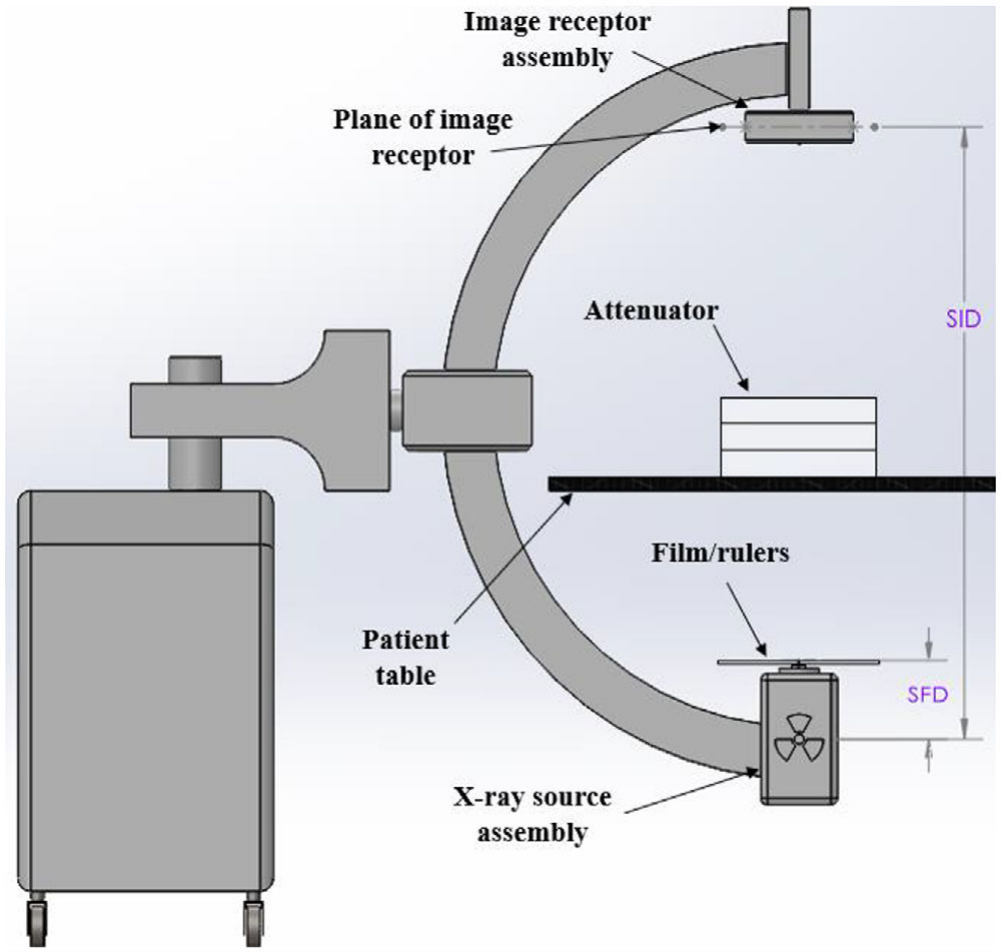
Typical setup for evaluating fluoroscopy collimator performance. The radiopaque rulers and film are located on the x‐ray source assembly. The attenuating media shown are three 30 cm × 30 cm × 5 cm acrylic slabs. In practice, any attenuating media can be used. The SFD and SID are shown. SFD, source‐to‐film/ruler distance; SID, source‐to‐image distance.

**FIGURE 2:**
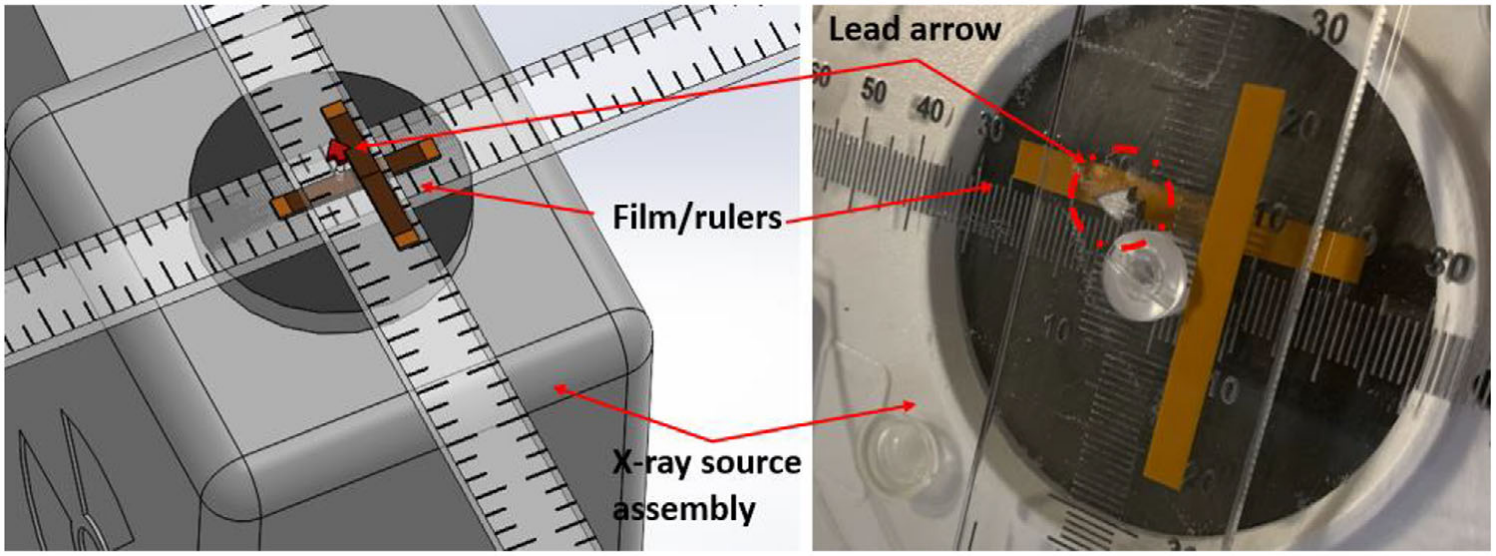
Rulers and film strips were placed over the x‐ray source assembly exit window. The plastic disk is the intersection point of the two rulers. Tape can be used to affix the ruler to the x‐ray source assembly cover if needed; in the depiction shown here and in Figure 1, the weight of the rulers holds the rulers to the x‐ray source assembly cover. The lead arrow is within the field and provides a reference dimension.

Figure 1 shows a typical setup for the fluoroscopic collimation evaluation test proposed in this work. Orthogonal radiopaque rulers (L‐Acryl‐400 mm‐LightField, Supertech, Elkhart, IN, US) with 1 mm indices are affixed to the x‐ray source assembly exit window (Figure 2). The ruler intersection must be located within the FOV. Two orthogonal radiochromic film strips (∼0.5 cm by 10 cm), cut from a larger film sheet (LD‐V1‐1012, Ashland Advanced Materials, Bridgewater NJ, USA), and a localizing marker are placed on the rulers (Figure 2). The film strips length must be larger than the radiation field size at the x‐ray source assembly exit window. The film used has a dose range of 2 cGy to 20 cGy; with the film located at the x‐ray source assembly exit window, the film is suitably exposed with 1 to 20 s of exposure for most fluoroscopy systems. Attenuating media are placed between the rulers/film and the image receptor to drive the system to a high technique allowing for rapid film exposure and preventing ghosting or burn‐in; usually, this media can be placed on the patient table as shown in Figure 1.

Two geometric parameters, the source‐to‐image distance (SID) and source‐to‐film/ruler distance (SFD) (Figure 1), are measured using manufacturer markings, the system display, and/or a calibrated ruler such as an optical distance indicator. The SID and SFD are used in Equation 1 for magnification correction of the ruler and film at the x‐ray source assembly exit window to the plane of the image receptor. In this work, a simplification is made in which the SFD is measured to the shortest source‐to‐skin distance (SSD) possible, and it is assumed rulers/film are located at this minimum SSD.

(1) $$M_{F\to \mathrm{DI}}=\frac{\mathrm{SID}}{\mathrm{SFD}}$$

With the test setup complete, fluoroscopic exposure is made until the radiochromic film perceptibly darkens. Exposure time can be modified by protocol selection and attenuators but is limited by maximum system output.

**FIGURE 3:**
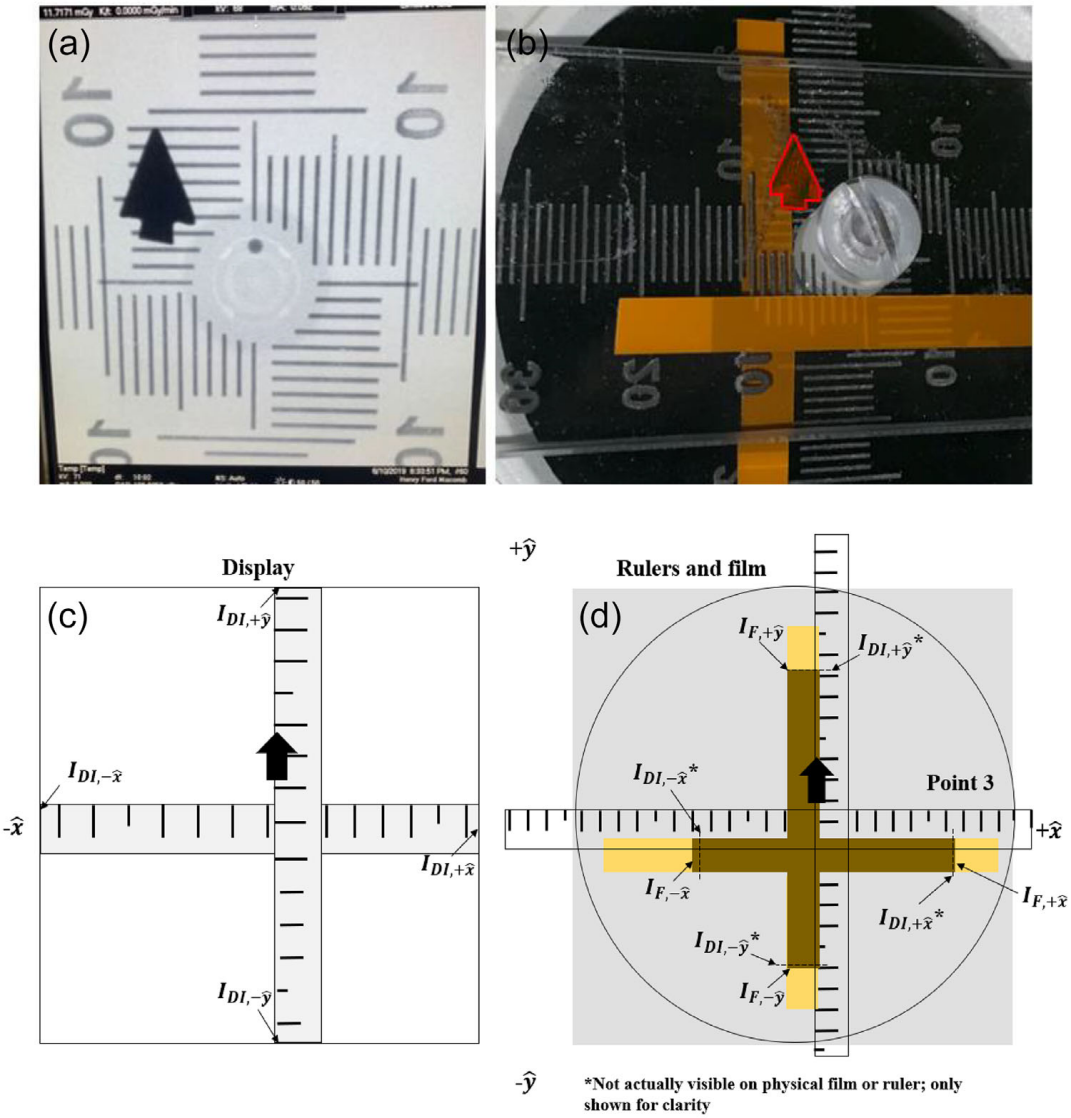
(a) Resultant image and (b) darkened film strips after fluoroscopy exposure. (a) and (b) appear slightly misaligned due to parallax. (a) and (b) are reproduced as (c) and (d), respectively, which additionally show the variable measured. For all measurements, the lead arrow marker ensures consistent orientation between the measurements made on the displayed image and the physical ruler.

The orthogonal ruler indices provide a single Cartesian frame of reference for all measurements; the ruler intersection (x,y)=(0mm,0mm) is assumed. On the displayed image (Figure 3a–c), the displayed image edges $I_{\mathrm{DI},i}$ are the ruler indices recorded at each edge $i=[-\hat{x},+\hat{y},+\hat{x},-\hat{y}]$ on the monitor. The radiation field edges $I_{F,i}$ are recorded as the ruler indices at the exposed/unexposed film line (Figure 3b–d). The lead marker identifies the orientation of the displayed image relative to the exposed film.

$I_{\mathrm{DI},i}$ and $I_{F,i}$ used to calculate the excess length (EL), excess width (EW), and the sum EL + EW of the radiation field relative to the displayed image as a percent of the individual system SID (%SID) using Equations 2 and 3. Note that $M_{F\to \mathrm{DI}}$ and the SID are not explicitly required to perform these calculations; however, they are required to determine the radiation and displayed field sizes for circular image receptor fluoroscopes manufactured on or after June 10 2006.

(2) $$\begin{array}{ccc} \mathrm{EW}\; & = & \frac{M_{F\to \mathrm{DI}}\times \left[\left(I_{F,\hat{x}}-I_{F,\;-\hat{x}}\right)-\left(I_{\mathrm{DI},\hat{x}}-I_{\mathrm{DI},\;-\hat{x}}\right)\right]}{\mathrm{SID}}\\ & & \times \;100\%=\frac{\left(I_{F,\hat{x}}-I_{F,\;-\hat{x}}\right)-\left(I_{\mathrm{DI},\hat{x}}-I_{\mathrm{DI},\;-\hat{x}}\right)}{\mathrm{SFD}}\times 100\% \end{array}$$

(3) $$\begin{array}{ccc} \mathrm{EL}\; & = & \frac{M_{F\to \mathrm{DI}}\times \left[\left(I_{F,\hat{y}}-I_{F,\;-\hat{y}}\right)-\left(I_{\mathrm{DI},\hat{y}}-I_{\mathrm{DI},\;-\hat{y}}\right)\right]}{\mathrm{SID}}\\ & & \times \;100\%=\frac{\left(I_{F,\hat{y}}-I_{F,\;-\hat{y}}\right)-\left(I_{\mathrm{DI},\hat{y}}-I_{\mathrm{DI},\;-\hat{y}}\right)}{\mathrm{SFD}}\times 100\% \end{array}$$

Because the SFD was assumed to be the minimum SSD, the values of EW and EL (and EW + EL) are slightly overestimated, which is conservatively safe; this is discussed in detail in Section 1 of the supplementary material. When implementing this method, the SFD can be easily measured to the film; however, if the SFD measured is greater than the actual SFD, even small inaccuracies can result in non‐identification of EW, EL, and EW + EL exceeding federal regulation. This is discussed in detail in Section 2 of the supplementary material.

To evaluate this method, fluoroscopy collimator performance evaluations were performed on 79 fluoroscopy systems (Table 1). All measurements were acquired at the maximum field size. Systems failed the performance evaluation if EL, EW, or EL + EW exceeded the regulatory limits specified in 21 CFR 1020.32^11^; passing criteria were EL and EW ≤ 3%SID and EL + EW ≤ 4%SID. These limits apply to all fluoroscopes except those with circular image receptors manufactured on or after June 10, 2006. For these fluoroscopes: (1) for maximum FOV ≤ 34 cm, 80% of the x‐ray field must overlap the displayed image; or (2) for maximum FOV > 34 cm, the x‐ray field cannot extend further than 2 cm past the displayed image. In this work, no distinction was made for image receptor shape; for all systems, the ratio of the elliptical area of the x‐ray field and the displayed image was calculated, assuming the total length and width of the x‐ray field and displayed image were the major and minor diameters, as appropriate. Any ratios < 0.80 were investigated. Additionally, $M_{F\to \mathrm{DI}}\times \left(I_{F,i}-I_{\mathrm{DI},i}\right)$ was computed for each edge i; any values > 2 cm were investigated.

**TABLE 1:** Manufacturers and models of evaluated fluoroscopy system.

System type	Total number	System types
Fixed C‐Arm	32	GE Innova 2100 ‐ 1 Philips Allura Xper ‐ 12 Philips Allura Clarity – 6 Philips Azurion – 1 Siemens Artis Axiom – 1 Siemens Artis Q – 4 Siemens Artis Zee – 7
Mini C‐Arm	24	GE OEC Elite MiniView – 2 Hologic Fluoroscan Insight 2 – 2 Hologic Fluoroscan Insight FD – 10 Orthoscan 1000 series – 4 Orthoscan FD – 6
Mobile C‐Arm	13	GE OEC Elite – 2 Medtronic O‐arm – 1 Philips BV Pulsera – 1 Philips Veradius Unity – 8 Siemens Cios Alpha – 1
Radiographic/ Fluoroscopic	10	GE Precision 500D – 1 Philips Diagnost – 2 Shimadzu Fluorospeed – 1 Shimadzu SonialVision – 2 Siemens Luminos – 2 Siemens Uroskop ‐ 2

Two types of uncertainty were calculated. The first is the uncertainty, *σ* (%SID), of the mean values of EW, EL, and EW + EL reported in Table 1 for each type of fluoroscopy system. A second systemic uncertainty is associated with the propagation of measurement error. An uncertainty of 0.5 mm was assumed for the SFD and each I term in Equations 2 and 3; the average uncertainty for EL, EW, and EL + EW was calculated as described in Part 3 of the supplementary material.

All individuals (two medical physicists, two medical physics residents, and two medical physics assistants) performing fluoroscopy EPEs provided the average time required to perform this method of collimator performance evaluation.

## 3 RESULTS

The results of the collimator performance evaluation for the 79 fluoroscopy systems are reported in Table 2. This table provides the mean, σ, and the maximum value of EW, EL, and EW + EL. Four systems were identified as failing: two mini C‐arms, one mobile C‐arm, and one radiographic/fluoroscopic system. One system (mini C‐arm) was found to have an area ratio < 0.80; this system also had EW and EL > 3%SID and EW + EL > 4%SID. One system (radiographic/fluoroscopic) was found to have one x‐ray field edge extend > 2 cm past the displayed image edge; this system also had EL > 3%SID.

**TABLE 2:** Average and maximum values for EW, EL, and (EW+EL).

Fixed C‐arm SID (cm) range: 90 to 130			
	EW	EL	(EW + EL)
Mean (%SID)	0.42%	0.32%	0.74%
*σ* (%SID)	0.43%	0.40%	0.59%
Maximum (%SID)	1.63%	0.95%	2.11%
Failed evaluation	0	0	0

Mini C‐arm SID (cm) range: 44 to 46			
	EW	EL	(EW + EL)
Mean (%SID)	1.32%	1.19%	2.50%
*σ* (%SID)	0.88%	0.94%	1.64%
Maximum (%SID)	4.44%	4.27%	8.71%
Failed evaluation	1	1	2

Mobile C‐arm SID (cm) range: 98 to 117			
	EW	EL	(EW + EL)
Mean (%SID)	0.74%	0.62%	1.37%
*σ* (%SID)	0.59%	0.70%	1.20%
Maximum (%SID)	2.20%	2.09%	4.27%
Failed evaluation	0	0	1

Radiographic/Fluoroscopic SID (cm) range: 102 to 124			
	EW	EL	(EW + EL)
Mean (%SID)	0.84%	0.74%	1.58%
*σ* (%SID) of mean	1.07%	0.61%	1.39%
Maximum (%SID)	3.67%	1.59%	5.00%
Failed evaluation	1	0	1

*Note*: *σ* is the standard deviation for the mean.

Abbreviations: EL, excess length; EW, excess width; SID, source‐to‐image distance.

The minimum and maximum measurement uncertainty due to the propagation of error for EW, EL, and EW + EL is provided in Table 3. The measurement error ranged from 0.11%SID to 0.95%SID for EW and EL and 0.16%SID to 1.35%SID for EW + EL. The measurement errors of EW and EL are very similar but not equal due to differences in the measured EW and EL for each system.

**TABLE 3:** Measurement uncertainty of EW, EL, and (EW + EL).

	Measurement uncertainty (%SID)				
	EW and EL		(EW + EL)		
System Type	Minimum	Maximum	Minimum	Maximum	SID (cm) range
Fixed‐C‐arm	0.26%	0.33%	0.36%	0.46%	90 to 130
Mini C‐arm	0.83%	0.95%	1.18%	1.35%	44 to 46
Mobile C‐arm	0.13%	0.48%	0.18%	0.68%	98 to 117
Radiographic/Fluoroscopic	0.11%	0.36%	0.16%	0.51%	102 to 124

Abbreviations: EL, excess length; EW, excess width; SID, source‐to‐image distance.

Table 4 provides the average factor of increased x‐ray exposure at the x‐ray source assembly exit window relative to the image receptor assembly cover, as calculated using the inverse square law. This factor ranged from 3.4 for radiographic/fluoroscopic systems with the x‐ray tube under the table to 14.6 for mini C‐arms.

**TABLE 4:** Average factor of increased x‐ray exposure at the x‐ray source assembly exit window relative to image receptor assembly cover.

System type	Factor of increased dose
Fixed C‐Arm	13.1±1.6
Mini C‐Arm	14.6±2.3
Mobile C‐Arm	12.4±2.5
Radiographic/Fluoroscopy (over table)	14.1±1.5
Radiographic/Fluoroscopy (under table)	3.4±0.4

The time required to set up and complete the fluoroscopy collimator performance evaluation described above ranged from 5 to 10 min.

## 4 DISCUSSION

Currently, collimator performance is quantitatively evaluated with the radiation field measuring device located on the image receptor cover.^6^, ^8^, ^9^, ^10^ This can result in challenges, including increased occupational dose, setting up time‐consuming or expensive visual recording devices, poor image quality if lead attenuators are used on the image receptor cover, and lengthy film exposure times. This note describes a method to evaluate collimator performance with the radiation field measurement device at the x‐ray source assembly exit window. This provides substantial benefits including quantification of radiation field size to displayed image size, more rapid film exposure, reproducibility among all systems, and potentially reduced time to assess collimator performance during an EPE.

Performing this method was simple as the radiation field and displayed image are measured after radiation exposure using a single common frame of reference with items commonly found in the imaging physicist's toolbox, noting that while radiochromic film is required, the cost per 0.5 cm × 10 cm strip using in this study was $0.33 USD. The film and rulers are easily placed in the radiation field and the measurements occur with the operator outside of the room, which can reduce occupational dose. Unlike placing lead sheets on the detector, attenuating media can be placed at any location between the x‐ray tube and image receptor to drive the x‐ray tube to a higher technique without impacting the positioning of the radiation field measurement device or the resultant image quality. The flexibility in the setup allowed for this method to evaluate 79 fluoroscopy systems of varied make and model.

This method increased radiation dose rate by a factor of up to 14.6 for film located on the x‐ray source assembly exit window relative to film assumed on the image receptor cover, decreasing the time required to sufficiently darken the film compared to conventional measurement techniques. This was invaluable for systems with limited maximum output; for example, the film visibly darkened after approximately 20 s of exposure at maximum output on a mini C‐arm compared to approximately 180 s of exposure required to darken film placed on the image receptor cover for the same system.

This method of fluoroscopy collimator performance evaluation has some limitations as implemented in this study. One limitation is that the field size was assessed only at the maximum FOV. The methodology presented here can be applied to all magnification modes without replacing film strips if care is taken not to overexpose the film, as the film will exhibit a stepped exposure pattern. Additionally, it may be challenging to perform the setup shown in Figure 1 for certain systems; for radiographic/fluoroscopic systems with under‐table tubes, the film/rulers must be placed on the table which reduces the benefit of increased air kerma rates.

The final and most important limitation is that error propagation can be problematic when using this method. The assumed uncertainty for the radiopaque ruler with 1 mm indices is 0.5 mm. If the ruler is located at the x‐ray source assembly exit window, measurement error propagation results in increased uncertainty as the SFD decreases. However, as demonstrated in Section 3 of the supplementary material, the effect of propagating measurement uncertainty is small in comparison to inaccurately measuring the SFD (Sections 1 and 2 of the supplementary material); small uncertainties in the measurement of the SFD may result in large under‐ or over‐estimations of EW, EL, and EW + EL. In this work, the SFD was assumed to be the minimum SSD which resulted in EW, EL, and EW + EL being overestimated; if these performance metrics did not pass, more care was taken in verifying measurement geometry to determine if the system required service. If the SFD is measured to each ruler/film strip, extreme care must be taken to ensure the accuracy of the measurement to prevent non‐identification of collimators not meeting performance requirements may be missed.

## 5 CONCLUSION

Fluoroscopy collimator performance is routinely assessed to ensure patient and staff ionizing radiation doses remain as low as reasonably achievable. A method for evaluating fluoroscopy system collimator performance was presented in which the radiation measurement device is placed at the x‐ray source assembly exit window. This geometry increased the exposure rate to the radiation field measurement device and utilized a single coordinate system for all measurements. This method meets the requirements of regulatory and accrediting bodies, provides quantifiable metrics that can be easily evaluated, and is simple to implement.

## AUTHOR CONTRIBUTIONS

Joseph R. Steiner—conception/design of method, data acquisition/analysis/interpretation, drafting/review of manuscript for important intellectual content, approval of the current submitted version, and agreement to be accountable for all aspects of the work. Courtney K. Morrison—design of method, data acquisition and results interpretation, minor drafting and substantial review of manuscript for important intellectual content, approval of the current submitted version, and agreement to be accountable for all aspects of the work. Mayur Vaya—substantial data acquisition and analysis, review of manuscript for important intellectual content, approval of the current submitted version, and agreement to be accountable for all aspects of the work. Nicholas Bevins—design of method, data acquisition/analysis/interpretation, review of manuscript for important intellectual content, approval of the current submitted version, and agreement to be accountable for all aspects of the work. Jeremy Christophel—substantial data acquisition and analysis, review of manuscript for important intellectual content, approval of the current submitted version, and agreement to be accountable for all aspects of the work. Matt Vanderhoek—design of method, data acquisition/analysis/interpretation, minor drafting and substantial review of manuscript for important intellectual content, approval of the current submitted version, and agreement to be accountable for all aspects of the work.

## CONFLICT OF INTEREST STATEMENT

The authors declare no conflicts of interest.

## Supporting information



Supporting Information
