# Supplementary material for: A new method to evaluate fluoroscopic system collimator performance
Source: J Appl Clin Med Phys. 2024 Oct 9;25(12):e14536. doi: 10.1002/acm2.14536 (PMC11633780; doi:10.1002/acm2.14536)
Supplement: Supplementary file 1 — Supporting Information [file ACM2-25-e14536-s001.docx]

# Title Page

Supplement for “Technical Note: A new method to evaluate fluoroscopic system collimator performance”

**Authors:**

Joseph R. Steiner^1*^, Courtney K. Morrison^2^, Mayur Vaya^2^, Nicholas Bevins^3^, Jeremy Christophel^2^, Matt Vanderhoek^2^

^1^Department of Radiology, University of Chicago, Chicago, IL 60637

^2^Department of Diagnostic Radiology and Nuclear Medicine, RUSH University Medical Center, Chicago IL 60612

^2^Department of Radiology, Henry Ford Health, Detroit, MI 48202

^3^Department of Radiology, Maine Medical Center, Portland, ME 04102

*Corresponding author: Contact at [jrsteiner@uchicago.edu](mailto:jrsteiner@uchicago.edu)

**Running title:**

Fluoroscopy Collimator Performance Evaluation

# Supplement Introduction

This document provides supplemental information regarding the manuscript: “Technical Note: A new method to evaluate fluoroscopic system collimator performance.” This supplement:

1. Clarifies the assumptions made in the manuscript regarding the SFD.
2. Evaluates the effect of small inaccuracies in the measurement of the $SFD.$
3. Analyzes the uncertainty inherent to the measurement process.

# Supplement Section 1: Clarification of assumptions

In the manuscript, it was assumed the $SFD$ was the minimum $SSD$ for three reasons. The first reason is that the $SSD$ will always be less than the actual distance of the source to the rulers/film. Thus, $EW$ and $EL$ (and similarly $EW+EL$) computed using Equations 2 and 3 with $SFD=SSD$ will be larger than if the true $SFD$ was used; this is conservatively safe as $EW$ and $EL$ will always be overestimated. The second reason is that the orthogonal rulers used in the work are overlapping; the $SFD$ is different for one ruler relative to the other. Choosing a conservatively safe approach that requires only one distance measurement (minimum $SSD$) greatly simplifies the procedure and reduces the likelihood of data input error. As described in Section 2, using a measured SFD greater than the actual SFD will underestimate $EW$, $EL$ and $EW + EL$ which can result in a system with $EW$ and/or $EL$ $> 3\%SID$ and/or $EW +EL > 4\%SID$ appearing to satisfy federal regulation. The third reason is that federal regulation specifies minimum $SSD$ for fluoroscopy systems; measuring the minimum $SSD$ satisfies this regulation.

A test case using typical geometry for a mini C-arm using the method described in the manuscript is used to demonstrate the effect of this assumption. For the orthogonal rulers used in this work, each arm has a thickness of 3 mm and the arms are separated by a 2 mm gap. In the worst case scenario, for one ruler arm $SFD=SSD + 3 mm$ and for the other ruler arm the $SFD=SSD + 8 mm$. In Figure S.1, the excess distances of 3 mm and 8 mm are represented as $\Delta z$. To compute $EW$ and $EL$, $I_{F}$ and $I_{DI}$ are measured along $\pm\hat{x}$ and $\pm\hat{y}$, and the $SSD$ was measured.


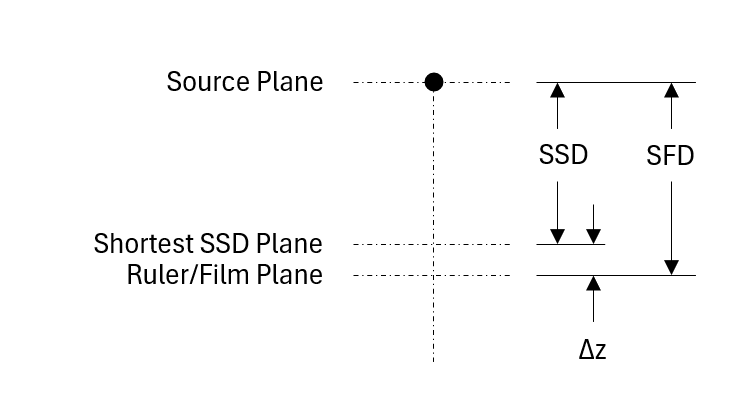


Figure S.1: Geometry showing the source plane, the SSD, the SFD, and Δz.

Equation S.1 can be used to calculate the overestimation $EW_{OE}$ of the $EW$, assuming $SFD$ equals $SSD$ (the assumption) and $SSD$ + $\Delta z$ where $\Delta z=3 mm$ or $8 mm$ and $EW$ is defined in Equation 2. The formalism for computing the overestimation $EL_{OE}$ of the $EL$ is the same, noting that if $EW_{OE}$ and $EL_{OE}$ are computed from a single test case, each will use a different $\Delta z$. For the purposes of this example, the minimum limits of $SSD$ specified in Federal Regulation 21CFR1020.32(g)(1,2) were utilized and are reported in Table S.1. The actual measured minimum $SSD$ is generally larger than these federal limits.

$$\begin{aligned} EW_{OE}=\left( \frac{\mathrm{EW}_{\mathrm{SSD}}}{EW_{SSD+\Delta z}}-1 \right)\times100\%=\left( \frac{\frac{\left( I_{F,\hat{x}}-I_{F, -\hat{x}} \right)-\left( I_{DI,\hat{x}}-I_{DI, -\hat{x}} \right)}{SSD}}{\frac{\left( I_{F,\hat{x}}-I_{F, -\hat{x}} \right)-\left( I_{DI,\hat{x}}-I_{DI, -\hat{x}} \right)}{SSD+\Delta z}}-1 \right)\times100\% \\ =\frac{\Delta z}{SSD}\times100\% \#\left( Eq. S.1 \right) \end{aligned}$$

| Table S.1: Percent by which $EW$ and $EL$ are increased by assuming the rulers/film occupy plane of the $SSD$ instead of $SSD+\Delta z$. | | | | | |  |
| --- | --- | --- | --- | --- | --- | --- |
|  |  |  |  |  |  |  |
|  |  |  |  |  |  |  |
| System Type | Minimum $SSD$ (cm) | $EW$ | | $EL$ | |  |
|  |  | $\Delta z$ (cm) | EW_OE_ | $\Delta z$ (cm) | EL_OE_ |  |
| Fixed C-arm | 20 | 0.3 | 1.5% | 0.8 | 4.0% |  |
| Mini C-arm (non-surgical) | 19 | 0.3 | 1.6% | 0.8 | 4.2% |  |
| Mini C-arm (surgical) | 10 | 0.3 | 3.0% | 0.8 | 8.0% |  |
| Mobile C-arm (non-surgical) | 30 | 0.3 | 1.0% | 0.8 | 2.7% |  |
| Mobile C-arm (surgical) | 20 | 0.3 | 1.5% | 0.8 | 4.0% |  |
| Rad/fluoro (non-surgical) | 38 | 0.3 | 0.8% | 0.8 | 2.1% |  |
| Rad/fluoro (surgical) | 20 | 0.3 | 1.5% | 0.8 | 4.0% |  |

The results of this calculation demonstrate that the values of $EW$ and $EL$ will be overestimated by 0.8% to 8.0% depending on the ruler orientation and the minimum SSD, which depends on system type. An overestimate of $EW$ or $EL$ by 8.0% is substantial and may result in false positives of collimators failing their performance evaluation; if $EW$ or $EL$ is calculated as $3\%SID$ (limit for failure) using this assumption, the true $EW$ or $EL$ would be $2.76\%SID$ which would satisfy federal regulation. For cases of failure when using this assumption, $EW$ and $EL$ can be more accurately calculated using careful measurement of the $SFD$ for each ruler arm to evaluate if the system needs service. This reduces the complexity for most systems that comply while providing the flexibility to perform more accurate measurements and calculations if needed.

# Supplement Section 2: The effect of small inaccuracies in the measurement of the $\boldsymbol{SFD}$

As shown in Supplement Section 1, measuring the $SFD$ as 3 mm or 8 mm less than the true $SFD$ resulted in overestimations of $EW$ and $EL$ ranging from 0.8% to 8.0%. By measuring the SFD to the actual film locations, a more accurate value of $EW$ and $EL$ (and the sum of those terms) can be achieved. However, caution must be exercised when measuring to the rulers, because if the $SFD$ measured ($SFD_{meas}$) and used for calculation is larger than the true $SFD$ ($SFD_{true}$), $EW$ and $EL$ will be underestimated, which may result in not identifying a fluoroscopy system collimator that does not satisfy the limits specified in Federal Regulation 21CFR1020.32. As the amount of under- or overestimation of $EW$ and $EL$ will be the same for a given $SFD_{true}$ and $SFD_{meas}$, two new variables are defined; $\Delta SFD=SFD_{true}-SFD_{meas}$ (which is equivalent to $\Delta z$ in Section 1) and $ED$ is the excess dimension which represents $EW$ and $EL$; $ED$ is calculated using Equation S.2 (only the calculation along $\hat{x}$ is shown). Note that Equation S.2 is the same as Equation S.1 where $SFD_{meas}=SSD$ and $SFD_{true}=SSD+\Delta z$, with a key difference being that in Equation S.1, $SFD_{meas}$ will always be less than or equal to $SFD_{true}$ because the rulers and film cannot be located nearer to the source than the minimum $SSD$.

$$\begin{aligned} ED=\left( \frac{\mathrm{ED}_{\mathrm{SFD}_{\mathrm{meas}}}}{\mathrm{ED}_{\mathrm{SFD}_{\mathrm{true}}}}-1 \right)\times100\%=\left( \frac{\frac{\left( I_{F,\hat{x}}-I_{F, -\hat{x}} \right)-\left( I_{DI,\hat{x}}-I_{DI, -\hat{x}} \right)}{SFD_{meas}}}{\frac{\left( I_{F,\hat{x}}-I_{F, -\hat{x}} \right)-\left( I_{DI,\hat{x}}-I_{DI, -\hat{x}} \right)}{SFD_{true}}}-1 \right)\times100\% \\ =\left( \frac{SFD_{true}}{SFD_{meas}}-1 \right)\times100\% \#\left( Eq. S.2 \right) \end{aligned}$$

Figure S.2 shows the under- and overestimation for $\Delta SFD$ ranging from -2 cm to 2 cm for $SFD_{true}=11.5 cm, 21.0 cm, \mathrm{and} 31.0 cm$, which are representative of mini-C-arms, surgical fluoroscopy systems, and general fluoroscopy systems. The same trends will occur with any $SFD_{true}$.

Figure S.2: Measured $ED$ over-and underestimation compared to the true $ED$ as a function of $\Delta SFD$ for $SFD_{true}=11.5 cm,21.0 cm,\mathrm{and}31.0 cm.$

As seen in Figure S.2, if $\Delta SFD$is negative, the calculated $ED$ will be less than the actual $ED$. In the worst case scenario shown by the chart, if $\Delta SFD = - 2.0 cm$, this underestimation will be $-17.4\%$, $-9.5\%$, and $-6.5\%$ for $SFD_{true}=11.5 cm$, $21.0 cm$, and $31.0 cm$, respectively. This can result in false negatives, where it appears the fluoroscopy system collimator performance satisfies federal regulation but in fact does not. This is the most important limitation of this method and extreme caution must be exercised if $SFD_{meas}$ is utilized for calculation of $EW$ and $EL$.

# Supplement Section 3: Analysis of measurement uncertainty

Finally, in the manuscript that two uncertainties were reported. The first uncertainty is $\sigma(\%SID)$, which the typical standard deviation of the mean value of $EW$, $EL$, and $EW + EL$ for each sample of measurements. $\sigma(\%SID)$ was reported solely as a comparative metric as it is unlikely the values of $EW$, $EL$, and $EW + EL$ are normal, as they are dependent on the state or condition of each fluoroscopy system collimator assessed. While other comparative metrics could be used (e.g., interquartile ranges), the value again is likely limited and the inclusion of this information may detract from the purpose of this manuscript, which is to outline a robust and simple method for evaluating fluoroscopy collimator performance.

The second uncertainty is the propagation of error from the measurement process. Reproducing Equation 2 and 3 from the manuscript as Equation S.3 and S.4 respectively

$$\begin{aligned} EW=\frac{\left( I_{F,\hat{x}}-I_{F, -\hat{x}} \right)-\left( I_{DI,\hat{x}}-I_{DI, -\hat{x}} \right)}{SFD}\times100\%\#\left( Eq. S.3 \right) \end{aligned}$$

$$\begin{aligned} EL=\frac{\left( I_{F,\hat{y}}-I_{F, -\hat{y}} \right)-\left( I_{DI,\hat{y}}-I_{DI, -\hat{y}} \right)}{SFD}\times100\%\#\left( Eq. S.4 \right) \end{aligned}$$

it is clear that if SFD has a constant measurement uncertainty (e.g., 0.5 mm), the uncertainty associated with the measurement will be larger if the rulers/film are located on the x-ray exit aperture ($\approx SSD$) relative to the image receptor cover ($\approx SID$); this is described below. Importantly, the analysis presented below is simplified to encompass only expected measurement error. The systemic errors associated with the assumption $SFD = SSD$ have been thoroughly described in Section 1 and Section 2 and are not included in this section.

To determine the amount of error associated with measurement, it is assumed all measured distances have an inherent uncertainty of $0.5 mm$. This is because the radiopaque rulers used have graduations of $1 mm$ and the measurement of the minimum $SSD$ was measured and/or reported with an accuracy of $1 mm$. Using typical propagation of error, the measurement uncertainty $\sigma_{EW}$ in $EW$ is calculated using the uncertainty of each measurement (indicated by $\sigma$ with the measurement variable as a subscript) as follows, noting the calculation of $EL$ will be equivalent. First, Equation S.3 can be rewritten as

$$EW=\frac{I_{F,\hat{x}}-I_{F, -\hat{x}}-I_{DI,\hat{x}}+I_{DI, -\hat{x}}}{SFD}\times100\%$$

which, if all uncertainties of all terms in the numerator are assumed to be the same ($\sigma_{I}$), results in an uncertainty of the numerator being

$$\sigma_{num}=\sqrt{\left( \sigma_{I_{F,\hat{x}}} \right)^{2}+\left( \sigma_{I_{F,-\hat{x}}} \right)^{2}+\left( \sigma_{I_{DI,\hat{x}}} \right)^{2}+\left( \sigma_{I_{DI,-\hat{x}}} \right)^{2}}=\sqrt{4\sigma_{I}^{2}}=2\sigma_{I}$$

The uncertainty of the denominator $SFD$ is simply $\sigma_{SFD}$.

Estimation of the uncertainty of $EW$ ($\sigma_{EW}$) is problematic because is it very likely that the numerator $I_{F,\hat{x}}-I_{F, -\hat{x}}-I_{DI,\hat{x}}+I_{DI, -\hat{x}}$ will be close to or equal to zero; thus the following methodology is used: A given function $z=z\left( x,y \right)$ has the associated uncertainty $\sigma_{z}=\sqrt{\left( \frac{\delta z}{\delta x}\cdot\sigma_{x} \right)^{2}+\left( \frac{\delta z}{\delta x}\cdot\sigma_{y} \right)^{2}}$. If $z=\frac{x}{y}$, the partial derivatives are $\frac{\delta z}{\delta x}=\frac{1}{y}$ and $\frac{\delta z}{\delta y}=-\frac{x}{y^{2}}$. With substitution

$$\sigma_{z}=\sqrt{\left( \frac{\sigma_{x}}{y} \right)^{2}+\left( -\frac{x\sigma_{y}}{y^{2}} \right)^{2}}$$

Using this derivation with the numerator and denominator for $EW$ as defined above,

$$\sigma_{EW}=\sqrt{\left( \frac{2\sigma_{I}}{SFD} \right)^{2}+\left( \frac{-\left( I_{F,\hat{x}}-I_{F, -\hat{x}}-I_{DI,\hat{x}}+I_{DI, -\hat{x}} \right)\times\sigma_{SFD}}{\left( SFD \right)^{2}} \right)^{2}}\times100\%$$

and thus the uncertainty associated with measurement is dependent upon the SFD, where a smaller SFD results in a larger relative uncertainty as %SID.

As a representative example, consider a mini-C-arm with $SFD=11.5 cm$, $\left( I_{F,\hat{x}}-I_{F, -\hat{x}} \right)-\left( I_{DI,\hat{y}}-I_{DI, -\hat{y}} \right)=0.25 cm$, and $SID = 44 cm$. $EW$ and $\sigma_{EW}$ are

$$EW=\frac{I_{F,\hat{x}}-I_{F, -\hat{x}}-I_{DI,\hat{x}}+I_{DI, -\hat{x}}}{SFD}\times100\%=\frac{2.5 mm}{115 mm}\times100\%=2.2\%SID$$

$$\sigma_{EW}=\sqrt{\left( \frac{2*0.5 mm}{115 mm} \right)^{2}+\left( \frac{-\left( 2.5 mm \right)\times0.5 mm}{\left( 115 mm \right)^{2}} \right)^{2}}\times100\%=0.87\%$$

As a comparative metric, consider if the $SFD$ is equal to $SID=440 mm$, which is similar to the case where the rulers/film are located on the image receptor cover. The numerator can be calculated as (noting that this is an estimate as it was not measured)

$$I_{F,\hat{x}}-I_{F, -\hat{x}}-I_{DI,\hat{x}}+I_{DI, -\hat{x}}=\frac{EW\times SFD}{100\%}=\frac{2.2\%\times440 mm}{100\%}=9.68 mm$$

and from this the uncertainty would be

$$\sigma_{EW}=\sqrt{\left( \frac{2*0.5 mm}{440 mm} \right)^{2}+\left( \frac{-\left( 9.68 mm \right)\times0.5 mm}{\left( 440 mm \right)^{2}} \right)^{2}}\times100\%=0.27\%$$

which is less than the uncertainty measured at the smaller $SFD$. This is a limitation of the methodology described in this manuscript; one must decide if the benefits of performing fluoroscopy collimation testing using the methodology in this manuscript outweigh the larger associated measurement uncertainty. Importantly, this measurement uncertainty is generally less than the error if the $SFD$ is not accurately measured (Section 2).

As one final note, the uncertainty of $EL$ is calculated in the same manner as $EW$, and the uncertainty of $EL+EW$ is calculated as

$$\sigma_{EL+EW}=\sqrt{\sigma_{EL}^{2}+\sigma_{EW}^{2}}$$

# Supplement Summary

This supplement illustrates the challenges associated with measurement error and uncertainty when assessing collimator performance using the method described in the manuscript. Anyone implementing this method for fluoroscopy collimator performance should note that performing measurements at a smaller $SFD$ will increase measurement uncertainty. However, the error associated with measuring the $SFD$ can result in large variation in the calculated values of $EW$, $EL$ and $EW+EL$. Measurement of the $SFD$ is the most critical aspect to ensure accuracy of this method, and the reader may consider assuming $SFD=SSD$. This assumption can result in more systems failing collimator evaluation; however in these specific cases more careful measurements of the $SFD$ can be performed to obtain more accurate calculated values. If the $SFD$ is overestimated, it is possible that systems failing collimator performance may be missed, and so extreme caution should be exercised when measuring the $SFD$.
